# Identification of immunogenic outer membrane proteins and evaluation of their protective efficacy against *Stenotrophomonas maltophilia*

**DOI:** 10.1186/s12879-018-3258-7

**Published:** 2018-07-27

**Authors:** Guangyang Xu, Xueping Tang, Xueyi Shang, Yan Li, Jing Wang, Junjie Yue, Yan Li

**Affiliations:** 1 No. 307 Hospital of PLA of Anhui Medical University, Hefei, 230032 China; 2 Department of Critical Care Medicine, Affiliated Hospital of Academy of Military Medical Sciences, Beijing, 100071 China; 3 Departmen of Respiratory Diseases, Taizhou Second People’s Hospital, Taizhou, 225500 Jiangsu China; 4 Beijing Institute of Biotechnology, Beijing, 100071 China

**Keywords:** Immunoproteomics, Smlt4123, *Stenotrophomonas maltophilia*, Vaccine candidate

## Abstract

**Background:**

*Stenotrophomonas maltophilia* (*S. maltophilia*) is an emerging global multiple-drug-resistant organism. It becomes increasingly challenging to treat *S. maltophilia* infection effectively. Novel therapeutic and preventive approaches targeting *S. maltophilia* infection are still lacking. This study aims to isolate outer membrane proteins (Omps) from *S. maltophilia* and use immunoproteomic technology to identify potential vaccine candidates of Omps against *S. maltophilia* infections.

**Methods:**

Omps from *S. maltophilia* culture were separated by two-dimensional electrophoresis and identified by matrix-assisted laser desorption/ionization time of flight mass spectrometry and nano liquid chromatography coupled fourier transform ion cyclotron resonance tandem mass spectrometry. Recombinant Omps were prepared and used to immunize mice, and the potency of mouse anti-Omp serum was tested in opsonophagocytic killing assay (OPKA). The effects of immunization with recombinant Omp on blood and tissue bacterial loads in a mouse model of *S. maltophilia*-induced infection were analyzed.

**Results:**

Outer membrane protein A (OmpA) and Smlt4123 were identified by mass spectrometry. Mouse anti-Smlt4123 serum significantly reduced the bacterial counts in healthy individuals’ blood in OPKA (*P* < 0.05) but mouse anti-OmpA serum did not. Enzyme-linked immunosorbent assay revealed that the antibody subtype of mouse anti-Smlt4123 antibody was IgG1. Eight hours after an intraperitoneal challenge with *S. maltophilia*, the bacterial loads in mouse blood were significantly lower in the mice receiving immunization with recombinant Smlt4123 than in the control mice receiving no immunization (*P* < 0.05), whereas the bacterial loads in other organs, such as the liver, spleen, lung, and kidney were similar in the two groups.

**Conclusions:**

The results revealed that the immunoproteomic approach was an efficient way to screen the immunogenic protein of *Stenotrophomonas maltophilia*. Moreover, the recombinant Smlt4123 had potential to protect mice from bacteremia caused by *S. maltophilia* in the early stages.

**Electronic supplementary material:**

The online version of this article (10.1186/s12879-018-3258-7) contains supplementary material, which is available to authorized users.

## Background

*Stenotrophomonas maltophilia* (*S. maltophilia*), a newly emerging global opportunistic Gram-negative pathogen, exists widely in nature and hospitals [1]. It can be isolated from soil, plant roots, and aqueous-associated sources, such as water treatment and distribution systems, tap water, and bottled water [2]. *S. maltophilia* strains of both clinical and environmental origin have been reported to adhere to abiotic surfaces and form bacterial biofilm [3, 4]. Besides, *S. maltophilia* can adhere to mouse tracheal mucus and thus cause respiratory tract infections [5]. As the third most frequently isolated nonfermentative Gram-negative bacilli following *Pesudomonas aeruginosa* and *Acinetobacter species* [6], *S. maltophilia* is also a newly emerging multiple-drug-resistant organism [2]. *S. maltophilia* can cause infections in multiple organs and tissues, such as the respiratory tract, biliary and urinary tract, skin, bone and joint, heart, brain, eyes, and soft tissues [7–9]. Risk factors for *S. maltophilia*-associated infections include immunosuppression, cancer, indwelling devices, mechanical ventilation, and broad spectrum antimicrobial therapy [10, 11]. The mortality rate of *S. maltophilia*-associated infection ranges from 14 to 69% in patients with bacteremia [12]. *S. maltophilia* has been found to show resistance to a broad array of antibiotics, such as trimethoprim-sulfamethoxazole (TMP-SMX), β-lactam antibiotics, macrolides, fluoroquinolones, cephalosporins, aminoglycosides, chloramphenicol, carbapenems, tetracyclines, and polymyxins [13–15]. Thus, it is becoming increasingly challenging for physicians to use conventional therapies to treat *S. maltophilia*-associated infections effectively. Novel therapeutic or preventive approaches targeting *S. maltophilia*-associated infections are greatly needed. Currently, vaccines or antibody based treatments have not been developed for *S. maltophilia*-associated infections.

Bacterial outer membrane proteins (Omps) play key roles in bacterial survival and multiplication in host systems. Omps of *Aeromonas salmonicida* have been found to regulate the adherence and serum and bile salt resistance of the bacteria [16]. Because many bacterial Omps can be easily recognized by the host immune system as foreign substances so to initiate host immune defense mechanism against the bacterial infection [17–20], they could be potential vaccine candidates against the infection. Immunoproteomic technology is now allowing screening immunogenic candidate proteins rapidly and effectively. In the current study, we used immunoproteomic technology to identify immunogenic Omps of *S. maltophilia*, developed antibodies against the Omps, and tested the effects of immunization with recombinant Omps on tissue bacterial loads in a mouse model of *S. maltophilia* infection.

## Methods

### Bacterial strain and human blood samples

The bacterial strain *S. maltophilia* K279a was used in this study. We chose this particular strain because the whole genome sequence of the bacterial strain is available and *S. maltophilia* K279a was considered as a representative genome sequence strain [21]. The bacteria were grown in lysogeny broth (LB) or on LB agar plates. *Escherichia coli* (*E. coli*) BL21 (DE3) were used for recombinant protein production. Blood samples from healthy individuals were obtained from the healthy volunteers, Affiliated Hospital of Military Medical Science in China.

### Animals and ethics

New Zealand White rabbits weighing approximately 2 kg and Female BALB/c mice (5–6 weeks old) were housed at the animal care center of the Academy of Military Medical Sciences (AMMS), China. All animals were maintained under specific pathogen-free conditions and were kept in a climate-controlled room (temperature of 22 ± 1 °C and humidity 55 ± 5%) with a 12 h light/dark cycle. Animals were sacrificed by euthanasia, and all experiments were handled according to protocols approved by Institutional Animal Care and Use Committee of AMMS. All efforts were made to minimize animal suffering.

### Rabbit anti-*S. maltophilia* serum preparation

*S. maltophilia* K279a were grown in LB at 37 °C shaker to reach an optical density at 600 nm (OD_600_) of 1.0 (approximately 4.0 × 10^8^ CFU/mL), and then inactivated by treatment with 0.15% (*v*/v) methanol at 37 °C for 24 h. The inactivated *S. maltophilia* was injected into two female New Zealand white rabbits (2 kg) (4.8 × 10^9^ CFU/time) with a two-week interval between injections. The first injection was emulsified with Freund’s complete adjuvant (Sigma), and the remaining two injections were emulsified with Freund′s incomplete adjuvant (Sigma). Freund’s complete adjuvant is considered to be the gold standard adjuvant for immunization [22]. The Freund’s complete adjuvant has been commonly used to induce antibody production in animal models [23]. Blood samples from the rabbits were collected 7 days before the first injection (pre-immune serum) and 10 days after the last injection (post-immune serum). An indirect enzyme-linked immunosorbent assay (ELISA) was used to measure antibody levels in the serum samples. Briefly, ELISA plates were coated with 10 μg/mL *S. maltophilia* total protein in coating buffer [0.05 M NaHCO_3_ (pH 9.6)] at 4 °C overnight and then blocked with 3% BSA in phosphate-buffered saline containing 0.1% Tween-20 (PBST) buffer for 1 h at 37 °C. The pre-immune serum (1:100 dilution in PBST) and post-immune serum (1:10240 dilution in PBST) were added to the wells. Horse radish peroxidase (HRP) conjugated anti-rabbit IgG secondary antibody was added. The color was developed by using the 3,3’,5,5’-Tetramethylbenzidine (TMB) single-component substrate solution (Beijing Solarbio Science & Technology Co., Ltd. China). The color development reaction was stopped by 100 μL 1 M H_2_SO_4_. The optical density at 450 nm (OD_450_) was determined in an ELISA plate reader.

### Preparation of outer membrane proteins (Omps)

*S. maltophilia* Omps were prepared according to the previous description [24]. In brief, *S. maltophilia* were grown in LB at 37 °C shaker to reach OD_600_ = 1.0 and then collected by centrifugation. The bacterial pellets were washed and re-suspended in ice cold TS buffer (150 mM NaCl, 10 mM Tris-HCl, pH 7.4). The bacterial suspension was kept in ice, sonicated for 20 min, and centrifuged at 6000 rpm/min for 10 min at 4 °C. The supernatant was transferred to a 10 mL centrifuge tube and further centrifuged at 12800 rpm/min for 30 min at 4 °C. The supernatant was discarded and the cell membrane pellets were re-suspended in 10 mL TS buffer by repeated pipetting. An equal volume of 2% Sarkosyl was added to the cell membrane suspension to dissolve the cytoplasmic membranes. The mixture was incubated at room temperature for 30 min with intermittent mixing, and then centrifuged at 12800 rpm/min for 30 min at 4 °C to collect Omps. The supernatant was discarded. The Omp pellets were re-suspended in 5 mL PBS. Protein concentration of the Omp suspension was determined by the Plus One 2-D Quant Kit (GE Healthcare).

### Two-dimensional polyacrylamide gel electrophoresis (2D PAGE) and western blot

Proteins in the Omp suspension were separated by 2D 10% sodium dodecyl sulfate polyacrylamide gel electrophoresis (SDS-PAGE) isoelectric focusing (IEF) electrophoresis. Two hundred μg Omps in 350 μL rehydration buffer [7 M Urea, 2 M Thiourea, 4% (*w*/*v*) CHAPS, 50 mM DTT] were loaded in pH 4–7 IPG strips (18 cm, GE Healthcare, USA). The loaded strips were rehydrated overnight and underwent electrophoresis at room temperature at 300 V for 1 h, 600 V for 1 h, and 1000 V for 1 h, and then 8000 V for 8 h, to reach a total of 64 kV­h. The proteins were separated and transferred to a polyvinylidene fluoride (PVDF) membrane (Bio-Rad, USA). The membrane was blocked with 5% skim milk in PBS overnight and then probed with the rabbit pre-immune or post-immune serum samples. The membrane was then washed and incubated with the secondary HRP-conjugated goat anti-rabbit IgG. The signals were developed by using the Super Signal West Dura Extended Duration Substrate (Pierce, USA), and the signals were detected using Image Scanner (GE Healthcare, USA).

### Matrix-assisted laser desorption/ionization time of flight mass spectrometry (MALDI-TOF-MS) and nano liquid chromatography coupled fourier transform ion cyclotron resonance tandem mass spectrometry (nanoLC-FT ICR MS/MS)

MALDI-TOF MS was performed on a Bruker Reflex III MALDI-TOF-MS (Bruker Daltonics, Germany) operating in the reflectron mode with 20 kV accelerating voltage and 23 kV Reflecting voltage [25]. A saturated solution of cyano-4-hydroxy-cinnamic acid in 50% acetonitrile and 0.1% trifluoroactic acid was used as the matrix. Sample preparation for the mass spectrometry followed the protocol reported by Geng and colleagues [25]. One μL the matrix solution and one μL sample solution were added in the Score 384 target well. The mass spectrometry analysis was performed by a specialist in the Instrument Application Center of the Academy of Military Medical Sciences following the previously published protocol [25]. In brief, mass accuracy for peptide mass finger-prints (PMF) analysis was calibrated with a 0.1–0.2 Da external standard, and internal calibration was carried out with enzyme autolysis peaks, at a resolution of 12,000. The nanoLC- FT ICR MS was performed on an APEX-Q FT-ICR tandem mass spectrometer (Bruker Daltonics, Germany) equipped with a 9.4 T superconducting magnet (Magnex Scientific, UK) and an infinity cell. The trypsin digested peptides were sequenced by auto MS mode with MS/MS boost function. The FT-ICR mass spectra were processed using Data Analysis 3.4 software (Bruker Daltonics GmbH, Germany) as a gateway to set up database searches.

### Expression and purification of recombinant Omps

To express OmpA and Smlt4123 proteins in *E. coli*, the Omp genes were first amplified by PCR and then cloned into the pET-30a (+) vector (Takara, Japan). The PCR primers were synthesized by Sangon Biotech (Shanghai, China) Co., Ltd. The primer sequences and cloning sites in pET-30a (+) are displayed in Additional file 1: Table S1. The recombinant vector was transformed into *E. coli* BL21 (DE3). The gene insertion in the recombinant vector was confirmed by DNA sequencing. The 6X-His tagged proteins expressed by the transformed *E. coli* BL21 were purified using Ni-agarose affinity chromatography according to the manufacturer instructions (GE Healthcare, USA). Endotoxin was removed from the Omp elution by using Triton-114 [26]. The Omp elution was analyzed by 10% SDS-PAGE.

### Preparation of mouse anti-Omp serum

Omp elution was injected into female BALB/c mice (6-week old) (5 μg/time) with a 2-week interval between injections. The first injection was emulsified with Freund’s complete adjuvant (Sigma), and the remaining two injections were emulsified with Freund′s incomplete adjuvant (Sigma). Blood samples from the retro-orbital plexus of anesthetized mice were collected 7 days before the first injection (pre-immune serum) and 10 days after the last injection (post-immune serum). An indirect ELISA was used to measure the antibody levels in the serum samples. The plates were coated with 10 μg/mL of Omp elution and then blocked with 3% BSA in PBST. The pre-immune and post-immune mouse serum samples were added to the wells. The secondary antibody, HRP-conjugated goat anti-mice IgG1 and IgG2a, were then added to the wells. The color was developed by TMB single-component substrate solution (Beijing Solarbio Science & Technology Co., Ltd. China). The plate was read at 450 nm in an ELISA plate reader.

### Opsonophagocytic killing assay

Opsonophagocytic killing assay (OPKA) was performed to determine the effectiveness of the anti-Omp serum in vitro. *S. maltophilia* were grown in LB at 37 °C shaker to reach OD_600_ 1.0. One mL of the bacterial culture was collected and centrifuges. The bacterial pellet was washed twice and re-suspended in 100 μL PBS (4.0 × 10^8^ CFU). The bacterial suspension was mixed with 50 μL mouse post-immune serum, for the control reaction, mouse pre-immune serum was added in place of the mouse post-immune serum. After incubation for 30 min at 37 °C, 350 μL blood samples from healthy individuals was added, and the mixture was rotated at 60 rpm at 37 °C for 1.5 h. Subsequently, 100 μL of the mixture were diluted with PBS and plated on LB agar. The plates were incubated at 37 °C overnight, and the number of colonies grown was counted on the next day.

### Immunization of mice with recombinant Omp

Female BALB/c mice (6-week old) were randomized into two groups (*n* = 10 in each group): Smlt4123-immuned group and control group. Mice in the Smlt4123-immuned group were received a subcutaneous immunization of 5 μg Smlt4123 in PBS and was emulsified with the same volume of Freund’s complete adjuvant (CFA, total volume 100 μL/mouse) for primary immunization and Freund’s incomplete adjuvant (IFA) for boosting on days 14 and 28. Mice in the control group were immunized similarly with a mixture of PBS and adjuvant. To establish a mouse infection model, mice were challenged with intraperitoneal infection of *S. maltophilia* two weeks after the final immunization. In our pilot study, we found that intraperitoneal inoculation of 10^7^ CFU *S. maltophilia* induced bacteremia in BALB/c mice, and inoculation of 10^9^ CFU resulted in an overwhelming infection, which caused mouse death within 24 h. Our pilot study also showed that the lethal dose of 50% for mice (LD_50_) was 5.8 × 10^8^ CFU (Additional file 1: Table S4). Thus, we used an intermediate dose, 4.0 × 10^8^ CFU, in the current study. *S. maltophilia* were grown in LB at 37 °C shaker to reach OD_600_ = 1.0. Cells were collected, washed twice with PBS, and re-suspended in PBS at the 2.0 × 10^9^ CFU/mL for the intraperitoneal injection. Mice in the Smlt4123-immuned group and the control group were injected intraperitoneally with 200 μL *S. maltophilia* suspension (4.0 × 10^8^ CFU) [27]. Eight hours after the injection, the mice were sacrificed by cervical dislocation. Mouse blood, liver, spleen, lung, and kidney were harvested and the tissues were homogenized in sterile PBS. Blood samples and homogenized tissue samples were cultured to determine bacterial burden.

### Statistical analyses

Continuous variables are presented as mean ± standard deviation (SD). Two-group comparison was analyzed using Student’s *t-* test. Wilcoxon signed rank test was used for paired comparisons and Mann Whitney test was used for unpaired comparisons. *P* value was 2-sided and *P* < 0.05 was considered statistically significant.

## Results

### Identification of Omps

**Fig. 1:**
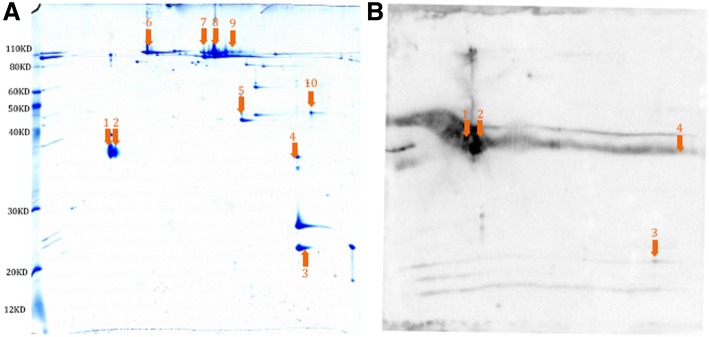
Two dimensional electrophoresis and Western blot analysis of *S. maltophilia* Omp preparation. **a** Image of the 2D gel. *S. maltophilia* Omps were prepared according to the description in the Methods. A total of 200 μg Omps was analyzed on 2D electrophoresis. After the electrophoresis, the 2D gel was stained with Coomassie Blue. All spots were analyzed by MALDI-TOF-MS and nanoLC-FT ICR MS/MS. Arrows of number 1 to 10 are pointing to the protein spots that had been identified by mass spectrometry. **b** Image of the Western blot. The 2D gel in (**a**) was probed with the rabbit anti-*S. maltophilia* serum. Protein spots 1 and 2 represent OmpA. Protein spots 3 and 4 represent Smlt4123. Protein spot 5 represents Omp TolC. Protein spots 6–9 represent TonB dependent receptor protein. Protein spot 10 represents fatty acid transport system membrane protein

Two dimensional (2D) gel electrophoresis of the Omp preparation showed several protein spots (Fig. 1a). All of the protein spots were analyzed by MALDI-TOF-MS and nanoLC-FT ICR MS/MS. The mass spectrometry analysis identified 5 proteins from 10 protein spots (Fig. 1a and Additional file 1: Table S2). The 5 proteins are OmpA (protein spot 1 and 2), outer membrane Omp family protein (Smlt4123) (protein spot 3 and 4), Omp TolC (protein spot 5), TonB dependent receptor (protein spot 6–9), and fatty acid transport system membrane protein (protein spot 10). OmpA and Smlt4123 genes were amplified by PCR and sequenced. The DNA sequences of the OmpA and Smlt4123 genes are displayed in Additional file 1: Table S3. Western blot analysis of the 2D gel revealed that OmpA had the strongest reaction to the rabbit anti-*S. maltophilia* serum whereas Smlt4123 reacted with the rabbit serum weakly (Fig. 1b). The rabbit pre-immune serum did not react with the protein spots on the 2D gel.

### Titer evaluation of mouse anti-Omp serum

**Fig. 2:**
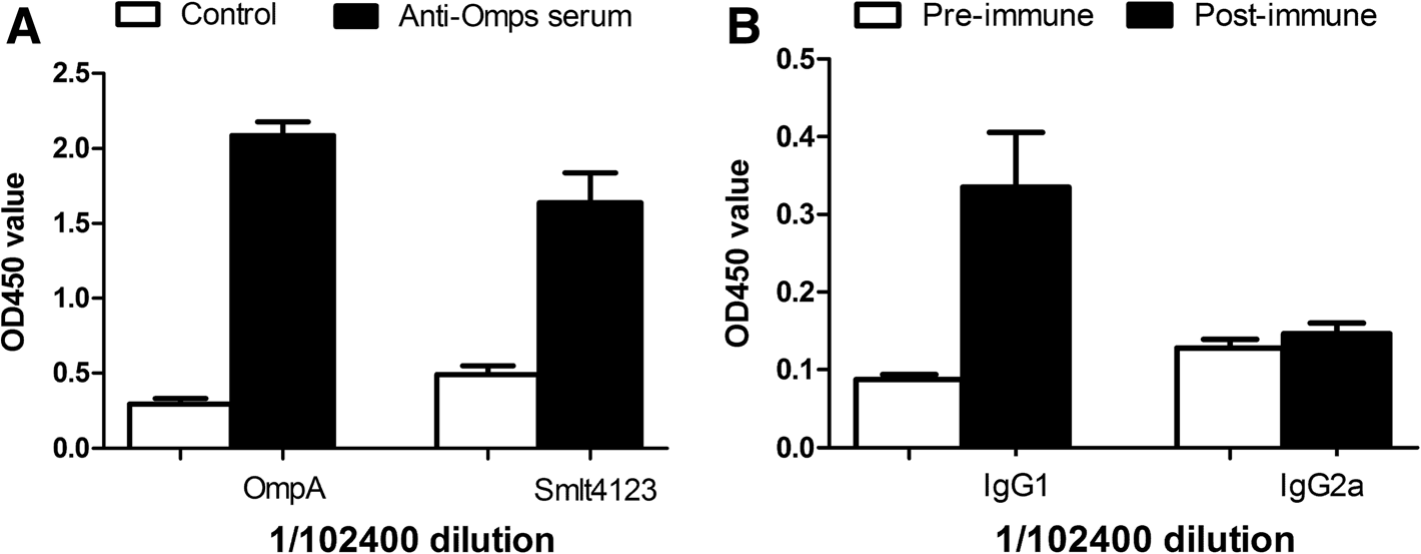
Titer evaluation of mouse anti-Omps serum. **a** Antibody levels in mouse anti-OmpA serum and mouse anti-Smlt4123 serum. Mice were injected with recombinant OmpA or Smlt4123 for three times (5 μg/time), and mice in the control group were injected with the elution buffer (*n* = 10/group). Two weeks after the final injection, serum were collected. The mouse serum was diluted at 1:102400 and analyzed by ELISA. Values were compared by Student’s *t*-test. *represents *P* < 0.05. **b** IgG1 and IgG2a levels in the pre-immune and post-immune mice injected with recombinant Smlt4123 (*n* = 8/group). Two weeks after the final immunization with recombinant Smlt4123, sera were collected. The mouse serum was diluted at 1:102400. Values were compared by paired Student’s *t*-test. * represents *P* < 0.05

Recombinant OmpA and Smlt4123 proteins produced from bacteria were used as antigen to immunize mice. ELISA showed that compared with the serum from control mice that were injected with the elution buffer, the mouse serum collected 2 weeks after the final immunization with the recombinant OmpA and Smlt4123 contained significantly increased levels of anti-OmpA and anti-Smlt4123 antibodies (*P* < 0.05, Fig. 2a). The serum was diluted at 1:102400 for ELISA, representing a titer greater than 1 × 10^5^ and also suggesting that OmpA and Smlt4123 appear to be powerful immunogens. In addition, we further identified the antibody subtype in the anti-Smlt4123 serum. Compared with the levels of IgG1 and IgG2a in the pre-immune serum, the IgG1 levels were significantly higher (*P* < 0.05), whereas the IgG2a levels were similar in the post-immune serum (Fig. 2b), indicating that anti-Smlt4123 antibody subtype may be IgG1 and recombinant Smlt4123 may induce Th2 immune response in the mice. We also identified the antibody subtype in the anti-OmpA serum. The result showed that compared with those in the pre-immune serum, the levels of both IgG1 and IgG2a were increased significantly after immunization with recombinant OmpA (Additional file 1: Figure S1).

### Potency evaluation of mouse anti-Omp serum

**Fig. 3:**
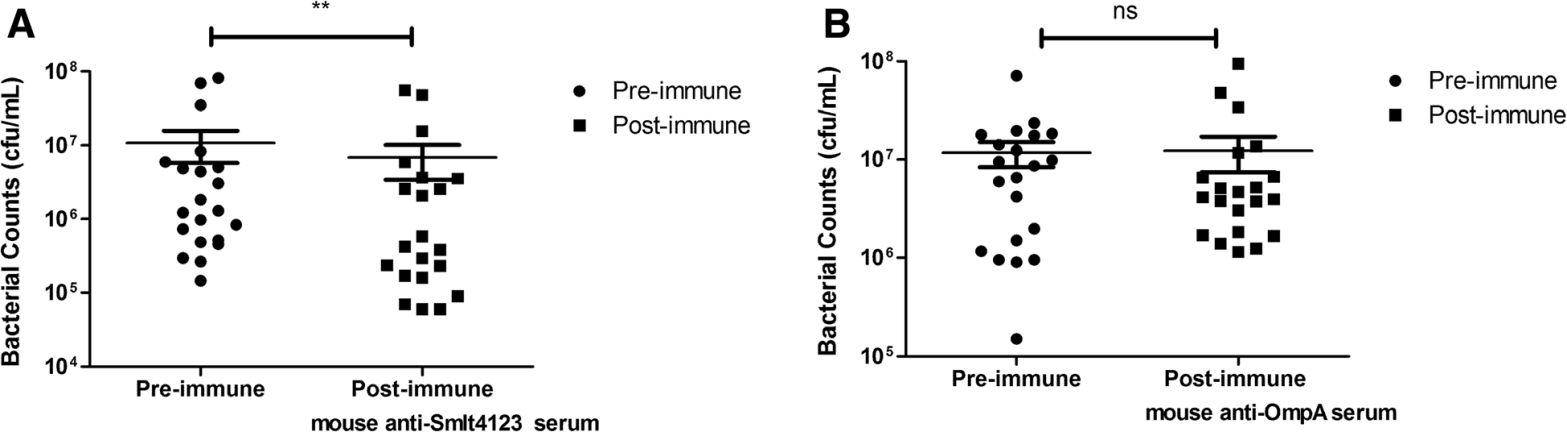
Opsonophagocytic killing assay of mouse anti-Omp serum. **a** Mouse anti-Smlt4123 serum significantly reduced bacterial counts in healthy individuals’ blood (*n* = 21). * represents *P* < 0.05. **b** Mouse anti-OmpA serum did not affect bacterial counts in healthy individuals’ blood (*n* = 21). ns: not significant. Paired Wilcoxon signed rank test was used to compare the effects of pre-immune serum versus post-immune serum on bacterial counts

OPKA was performed to evaluate the potency of the mouse anti-Omp serum in vitro. The mouse anti-Smlt4123 serum significantly reduced *S. maltophilia* counts in healthy individuals’ blood (*P* < 0.05, Fig. 3a). In contrast to the anti-Smlt4123 serum, the mouse anti-OmpA serum did not affect *S. maltophilia* counts in the blood of healthy individuals (Fig. 3b). These data suggest that mouse anti-Smlt4123 antibody may have some protective effects against *S. maltophilia* infection. We used the Smlt4123 protein sequence as a query and the Basic Local Alignment Search Tool to search genome sequences of 11 other *S. maltophilia* strains. Of the 11 strains, 4 have a protein with 100% amino acid sequence similarity to the Smlt4123 sequence; 6 have a protein showing > 90% amino acid sequence similarity to the Smlt4123 sequence; one showed no homologue. These results suggest that Smlt4123 may be highly conserved among *S. maltophilia* strains.

### Assessment of protective effects of immunization with recombinant Smlt4123 against *S. maltophilia* infection

**Fig. 4:**
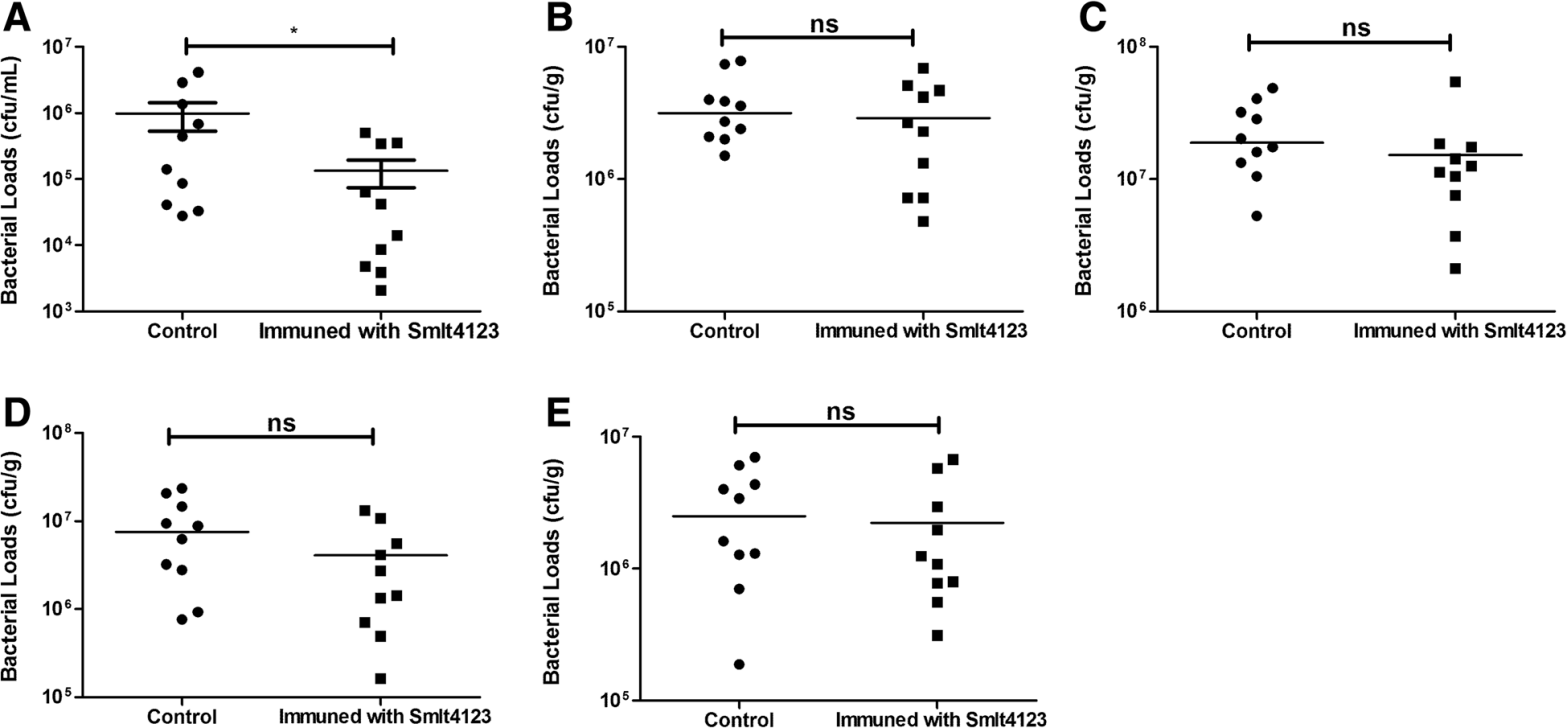
Effects of immunization of recombinant Smlt4123 on tissue bacterial loads in a mouse model of *S. maltophilia*-induced infection. Immunization with recombinant Smlt4123 significantly reduced the bacterial loads in the blood (**a**), but had no effects on the bacterial loads in other organs, including the liver (**b**), spleen (**c**), lung (**d**), and kidney (**e**). Blood (*n* = 10/group), and organs (*n* = 10/group) were collected from the vaccinated and control mice 8hours after the infection with *S. maltophilia* (4.0 × 10^8^ CFU). Unpaired Mann Whitney test was used for the comparison. * represents *P* < 0.05

We then tested the protective effects of immunization with recombinant Smlt4123 on bacterial loads in a mouse model of *S. maltophilia*-induce infection. The bacterial loads in the blood, liver, spleen, lung, and kidney were compared in the control mice versus the mice that were immunized with recombinant *S. maltophilia* Smlt4123. Eight hours after infection with 4.0 × 10^8^ CFU *S. maltophilia*, bacterial loads in the blood were significantly lower in the vaccinated mice than in the control mice, whereas bacterial loads in the other organs were similar between the two groups (Fig. 4).

## Discussion

Omps are highly abundant proteins of Gram-negative pathogens and can interact with the host immune system directly. In the current study, we used mass spectrometry and immunoproteomic technology to screen immunogenic Omps of *S. maltophilia* and successfully identified Smlt4123 and OmpA as immunogenic proteins.

Previous studies have demonstrated that bacterial Omps can be promising vaccine candidates against bacterial infection. Hamid and Jain have identified a strong immunogenic Omp from *Salmonella enterica serovar Typhimurium* and demonstrated that the Omp can induce humoral and cell-mediated immune responses in rats [28]. They have also found that the vaccination of the Omp can protect rats against typhoid [28]. McConnell and colleague have developed an Omp complex vaccine containing multiple bacterial membrane antigens of *Acinetobacter baumannii* [18]. They have tested the Omp complex vaccine in a murine sepsis model and found that the vaccine can evoke humoral and cellular responses, decrease post-infection bacterial loads and serum levels of pro-inflammatory cytokines, and protect mice from *Acinetobacter baumannii* infection [18]. Recently, Olsen and colleague have developed a vaccine construct based on the VD4s region from the *Chlamydia trachomatis* major outer membrane protein and demonstrated that the vaccine construct can protect mice against *Chlamydia trachomatis* challenge, reduce bacterial loads in mouse vagina, and prevent pathogenesis in mouse upper genital tract [29]. Wang and colleagues have shown that Omps are involved in inducing host defense against *S. maltophilia* infection in channel catfish [30].

OmpA is abundantly expressed in *Edwardsiella tarda* and highly immunogenic in rabbit and fish, and vaccination of common carp with recombinant OmpA protects the fish from *Edwardsiella tarda* infection and increases the fish survival [20]. In contrast to the report by Maiti and colleagues [20], the current study found that mouse anti-recombinant OmpA serum did not affect *S. maltophilia* counts in the OPKA although the recombinant OmpA appeared to be strongly immunogenic in mice. However, whether the recombinant OmpA could protect mice from *S. maltophilia* challenge remains unclear. Further investigation is required to clarify whether OmpA could also be a potential vaccine candidate against *S. maltophilia* infection.

The current study shows that vaccination with recombinant Smlt4123 significantly reduced post-infection bacterial loads in mouse blood but did not affect the bacterial loads in mouse organs 8 h after the mice were challenged with *S. maltophilia*. The relatively short-time exposure to the *S. maltophilia* challenge may explain why the vaccination with recombinant Smlt4123 only reduced the bacterial loads in mouse blood but not in other organs. It is possible that we could observe reduced bacterial loads in the tissues of liver, spleen, lung, and kidney in mice immunized with recombinant Smlt4123 if we could extend the observation time longer than 8 h after the infection challenge. Previous studies have showed that bacterial loads in blood and organs of mice immunized with Omp were decreased significantly at post-infection 12, 14, 24, or 48 h compared with those of the mice without immunization [19, 31, 32]. Therefore, we determined bacterial loads in liver, spleen, lung, and kidney of PBS immunized and Smlt4123 immunized mice at different time points. The result showed that 4, 8, 24 h post-infection no differences in bacterial loads were observed between groups of mice in these tissues (Additional file 1: Figure S2). Moreover, the use of recombinant Smlt4123 as a single antigen may have limited vaccination efficacy. A previous study has shown that Omp complex vaccine appears to reduce the number of *Acinetobacter baumannii* in multiple mouse organs [18]. Stranger-Jones and colleagues have also shown that individual vaccination with *Staphylococcus aureus* surface antigen confers only partial protection from infection, whereas vaccination with a combination of multiple antigens protects mice from bacterial challenge completely [33].

In this study, we found that mouse anti-recombinant Smlt4123 serum significantly reduced *S. maltophilia* counts in the OPKA. This result indicated that the mouse anti-recombinant Smlt4123 serum enhanced opsonophagocytic killing activity of blood. We also showed that mice receiving vaccination with recombinant Smlt4123 had significantly lower *S. maltophilia* loads in the blood than mice without the vaccination, whereas no differences in bacterial loads were observed between groups of mice in liver, spleen, lung, and kidney. This result suggested that immunization of mouse with rSmlt4123 could provide a protection from bacteremia caused by *S. maltophilia* in the early stages.

## Conclusions

Our findings indicated that the immunoproteomic approach was an efficient way to screen the immunogenic protein of *S. maltophilia*. Besides, we found that the mouse anti-recombinant Smlt4123 serum enhanced opsonophagocytic killing activity of blood in vitro. Moreover, the recombinant Smlt4123 had potential to protect mice from bacteremia caused by *S. maltophilia* in the early stages.

## Additional file

Additional file 1: **Table S1.** Primers used for cloning. Underlining indicates the recognition sites of restriction enzymes. **Table S2.** Identification of protein spots on 2D gel. Proteins identified by MALDI-TOF-MS and nanoLC-FT ICR MS/MS from *S. maltophilia* OMPs shown in Fig. 1a. **Table S3.** DNA sequences of Smlt0955 and Smlt4123 of *S. maltophilia.* OmpA and Smlt4123 genes were amplified by PCR and sequenced. **Table S4.** Determination of LD50. Fifty percent lethal dose (LD50) value of *S.maltophilia* to BALB/c mice was tested. **Figure S1.** IgG1 and IgG2a levels in the pre-immune and post-immune mice injected with recombinant OmpA. Two weeks after the final immunization with recombinant OmpA, serum were collected. The pre-immune and post-immune mouse serum were diluted at 1:10000 and analyzed by ELISA. Values were compared by paired Student’s *t*-test. **Figure S2.** Bacterial loads in liver, spleen, lung, kidney of mice at different time points. Organs were collected from the PBS immunized and Smlt4123 immunized mice 4, 8, 24 h post-infection, and bacterial loads in liver, spleen, lung, kidney of the vaccinated and control mice were determined. However, no differences in bacterial loads were observed between groups of mice in the liver, spleen, lung, and kidney. Unpaired Mann Whitney test was used for the comparison. ns represents *P* > 0.05. (DOCX 309 kb)

## Abbreviations

2D PAGE: Two-Dimensional Polyacrylamide Gel Electrophoresis
ELISA: Enzyme-linked immunosorbent assay
LB: Lysogeny broth
MALDI-TOF-MS: Matrix-assisted laser desorption/ionization time of flight mass spectrometry
Omps: Outer membrane proteins
PMF: Peptide mass finger-prints

## References

[1] Looney WJ, Narita M, Muhlemann K (2009). Stenotrophomonas maltophilia: an emerging opportunist human pathogen. Lancet Infect Dis.

[2] Brooke JS (2012). Stenotrophomonas maltophilia: an emerging global opportunistic pathogen. Clin Microbiol Rev.

[3] Lai CH, Wong WW, Chin C, Huang CK, Lin HH, Chen WF, Yu KW, Liu CY (2006). Central venous catheter-related Stenotrophomonas maltophilia bacteraemia and associated relapsing bacteraemia in haematology and oncology patients. Clin Microbiol Infect.

[4] O'Donnell MJ, Tuttlebee CM, Falkiner FR, Coleman DC (2005). Bacterial contamination of dental chair units in a modern dental hospital caused by leakage from suction system hoses containing extensive biofilm. J Hosp Infect.

[5] Zgair AK, Chhibber S (2011). Adhesion of Stenotrophomonas maltophilia to mouse tracheal mucus is mediated through flagella. J Med Microbiol.

[6] Hotta G, Matsumura Y, Kato K, Nakano S, Yunoki T, Yamamoto M, Nagao M, Ito Y, Takakura S, Ichiyama S (2014). Risk factors and outcomes of Stenotrophomonas maltophilia bacteraemia: a comparison with bacteraemia caused by Pseudomonas aeruginosa and Acinetobacter species. PLoS One.

[7] Platsouka E, Routsi C, Chalkis A, Dimitriadou E, Paniara O, Roussos C (2002). Stenotrophomonas maltophilia meningitis, bacteremia and respiratory infection. Scand J Infect Dis.

[8] Sakhnini E, Weissmann A, Oren I (2002). Fulminant Stenotrophomonas maltophilia soft tissue infection in immunocompromised patients: an outbreak transmitted via tap water. Am J Med Sci.

[9] Falagas ME, Kastoris AC, Vouloumanou EK, Dimopoulos G (2009). Community-acquired Stenotrophomonas maltophilia infections: a systematic review. Eur J Clin Microbiol Infect Dis.

[10] Paez JI, Costa SF (2008). Risk factors associated with mortality of infections caused by Stenotrophomonas maltophilia: a systematic review. J Hosp Infect.

[11] Metan G, Hayran M, Hascelik G, Uzun O (2006). Which patient is a candidate for empirical therapy against Stenotrophomonas maltophilia bacteraemia? An analysis of associated risk factors in a tertiary care hospital. Scand J Infect Dis.

[12] Lai CH, Chi CY, Chen HP, Chen TL, Lai CJ, Fung CP, Yu KW, Wong WW, Liu CY (2004). Clinical characteristics and prognostic factors of patients with Stenotrophomonas maltophilia bacteremia. J Microbiol Immunol Infect.

[13] Li XZ, Zhang L, McKay GA, Poole K (2003). Role of the acetyltransferase AAC(6’)-Iz modifying enzyme in aminoglycoside resistance in Stenotrophomonas maltophilia. J Antimicrob Chemother.

[14] Zhao Y, Niu W, Sun Y, Hao H, Yu D, Xu G, Shang X, Tang X, Lu S, Yue J (2015). Identification and characterization of a serious multidrug resistant Stenotrophomonas maltophilia strain in China. Biomed Res Int.

[15] Al-Hamad A, Upton M, Burnie J (2009). Molecular cloning and characterization of SmrA, a novel ABC multidrug efflux pump from Stenotrophomonas maltophilia. J Antimicrob Chemother.

[16] Ebanks RO, Goguen M, McKinnon S, Pinto DM, Ross NW (2005). Identification of the major outer membrane proteins of Aeromonas salmonicida. Dis Aquat Org.

[17] Jang IJ, Kim IS, Park WJ, Yoo KS, Yim DS, Kim HK, Shin SG, Chang WH, Lee NG, Jung SB (1999). Human immune response to a Pseudomonas aeruginosa outer membrane protein vaccine. Vaccine.

[18] McConnell MJ, Domínguez-Herrera J, Smani Y, López-Rojas R, Docobo-Pérez F, Pachón J (2011). Vaccination with outer membrane complexes elicits rapid protective immunity to multidrug-resistant Acinetobacter baumannii. Infect Immun.

[19] Luo G, Lin L, Ibrahim AS, Baquir B, Pantapalangkoor P, Bonomo RA, Doi Y, Adams MD, Russo TA, Spellberg B (2012). Active and passive immunization protects against lethal, extreme drug resistant-Acinetobacter baumannii infection. PLoS One.

[20] Maiti B, Shetty M, Shekar M, Karunasagar I, Karunasagar I (2011). Recombinant outer membrane protein a (OmpA) of Edwardsiella tarda, a potential vaccine candidate for fish, common carp. Microbiol Res.

[21] Crossman LC, Gould VC, Dow JM, Vernikos GS, Okazaki A, Sebaihia M, Saunders D, Arrowsmith C, Carver T, Peters N (2008). The complete genome, comparative and functional analysis of Stenotrophomonas maltophilia reveals an organism heavily shielded by drug resistance determinants. Genome Biol.

[22] Colavecchia SB, Jolly A, Fernandez B, Fontanals AM, Fernandez E, Mundo SL (2012). Effect of lipoarabinomannan from Mycobacterium avium subsp avium in Freund's incomplete adjuvant on the immune response of cattle. Braz J Med Biol Res.

[23] Rajput ZI, Hu SH, Xiao CW, Arijo AG (2007). Adjuvant effects of saponins on animal immune responses. J Zhejiang Univ Sci B.

[24] Carlone GM, Thomas ML, Rumschlag HS, Sottnek FO (1986). Rapid microprocedure for isolating detergent-insoluble outer membrane proteins from Haemophilus species. J Clin Microbiol.

[25] Geng H, Zhu L, Yuan Y, Zhang W, Li W, Wang J, Zheng Y, Wei K, Cao W, Wang H (2008). Identification and characterization of novel immunogenic proteins of Streptococcus suis serotype 2. J Proteome Res.

[26] Aida Y, Pabst MJ (1990). Removal of endotoxin from protein solutions by phase separation using triton X-114. J Immunol Methods.

[27] Rouf R, Karaba SM, Dao J, Cianciotto NP (2011). Stenotrophomonas maltophilia strains replicate and persist in the murine lung, but to significantly different degrees. Microbiology.

[28] Hamid N, Jain SK (2008). Characterization of an outer membrane protein of Salmonella enterica serovar typhimurium that confers protection against typhoid. Clin Vaccine Immunol.

[29] Olsen AW, Follmann F, Erneholm K, Rosenkrands I, Andersen P (2015). Protection against chlamydia trachomatis infection and upper genital tract pathological changes by vaccine-promoted neutralizing antibodies directed to the VD4 of the major outer membrane protein. J Infect Dis.

[30] Wang X, Peng L, Wang K, Wang J, He Y, Wang E, Chen D, Ouyang P, Geng Y, Huang X (2016). The outer membrane proteins of Stenotrophomonas maltophilia are potential vaccine candidates for channel catfish (Ictalurus punctatus). Fish Shellfish Immunol.

[31] Huang W, Wang S, Yao Y, Xia Y, Yang X, Long Q, Sun W, Liu C, Li Y, Ma Y (2015). OmpW is a potential target for eliciting protective immunity against Acinetobacter baumannii infections. Vaccine.

[32] Singh R, Capalash N, Sharma P (2017). Immunoprotective potential of BamA, the outer membrane protein assembly factor, against MDR Acinetobacter baumannii. Sci Rep.

[33] Stranger-Jones YK, Bae T, Schneewind O (2006). Vaccine assembly from surface proteins of Staphylococcus aureus. Proc Natl Acad Sci U S A.

