# Supplementary material for: Identification of immunogenic outer membrane proteins and evaluation of their protective efficacy against Stenotrophomonas maltophilia
Source: BMC Infect Dis. 2018 Jul 27;18:347. doi: 10.1186/s12879-018-3258-7 (PMC6062925; doi:10.1186/s12879-018-3258-7)
Supplement: Supplementary file 1 — Table S1. Primers used for cloning. Underlining indicates the recognition sites of restriction enzymes. Table S2. Identification of protein spots on 2D gel. Proteins identified by MALDI-TOF-MS and nanoLC-FT ICR MS/MS from S. maltophilia OMPs shown in Fig. 1a. Table S3. DNA sequences of Smlt0955 and Smlt4123 of S. maltophilia. OmpA and Smlt4123 genes were amplified by PCR and sequenced. Table S4. Determination of LD50. Fifty percent lethal dose (LD50) value of S.maltophilia to BALB/c mice was tested. Figure S1. IgG1 and IgG2a levels in the pre-immune and post-immune mice injected with recombinant OmpA. Two weeks after the final immunization with recombinant OmpA, serum were collected. The pre-immune and post-immune mouse serum were diluted at 1:10000 and analyzed by ELISA. Values were compared by paired Student’s t-test. Figure S2. Bacterial loads in liver, spleen, lung, kidney of mice at different time points. Organs were collected from the PBS immunized and Smlt4123 immunized mice 4, 8, 24 h post-infection, and bacterial loads in liver, spleen, lung, kidney of the vaccinated and control mice were determined. However, no differences in bacterial loads were observed between groups of mice in the liver, spleen, lung, and kidney. Unpaired Mann Whitney test was used for the comparison. ns represents P > 0.05. (DOCX 309 kb) [file 12879_2018_3258_MOESM1_ESM.docx]

**Additional file 1**

**Table S1 Primers used for cloning**

| **Gene name** | **Forward primer (5’-3’)** | **Reverse primer (5’-3’)** | **Restriction enzymes** |
| --- | --- | --- | --- |
| Smlt0955 | CGGAATTCCAGGAGTTCGATGACCGC | CCCTCGAGTTAGTTCTGGACGTTCAGC | EcoRI, XhoI |
| Smlt4123 | CGGAATTCCAGGACAGCAGCTCCACCGAT | CCCTCGAGTCAGAAGCGGGCACCGAT | EcoRI, XhoI |

**Table S2 Identification of protein spots on 2D gel**

| **Spot No. on gel** | **Accession** | **Mass** | **T Score** | **Sequence coverage%** | **Description** |
| --- | --- | --- | --- | --- | --- |
| 1 | gi\|190572988 | 39467 | 166 | 37 | outer membrane protein A |
| 2 | gi\|190572988 | 39467 | 174 | 40 | outer membrane protein A |
| 3 | gi\|190575954 | 22581 | 142 | 22 | outer membrane Omp family protein |
| 4 | gi\|190575954 | 22581 | 116 | 22 | outer membrane Omp family protein |
| 5 | gi\|190575774 | 48678 | 220 | 70 | outer membrane protein TolC |
| 6 | gi\|190575751 | 106360 | 339 | 36 | TonB dependent receptor protein |
| 7 | gi\|190572429 | 1031109 | 392 | 42 | TonB dependent receptor protein |
| 8 | gi\|190575314 | 101905 | 417 | 42 | TonB dependent receptor protein |
| 9 | gi\|190575314 | 101905 | 144 | 16 | TonB dependent receptor protein |
| 10 | gi\|190572491 | 49950 | 164 | 51 | Fatty acid transport system membrane protein |

**Table S3 DNA sequences of Smlt0955 and Smlt4123 of S. *maltophilia***

| Protein | Gene | NCBI Reference Sequence | DNA sequence |
| --- | --- | --- | --- |
| OmpA | Smlt0955 | WP_005408234 | ATGAACAAGAAGATCCTTACTGCCGCGCTGCTGGGTGGTCTGGCTTTCGCCCAGGCTGCGTCCGCGCAGGAGTTCGATGACCGCTGGTACCTGACCGGTTCGGCCGGCTTCAACTTCCAGGACAGCGACCGCCTGACCAATGACGCTCCGTTCGTCACCCTGGGCCTGGGCAAGTTCATCAGCCCGAACTGGTCGCTGGACGGTGAGCTGAACTACCAGAACCCGAACTTCGACGCCAACAAGGACATGAACTGGTCGCAGTACGGCGTCTCGCTGGACCTGCGTCGCCACTTCATCAAGGAAGGCCGCGGCTGGAACCCGTACCTGCTGGCCGGCCTGGGCTACCAGAAGTCGGAAGAAGAGTACAACCCGATCAGCGGTGGCCTGGCTGACCGCAAGGACGGCAACTTTGCCGCCAAGGTCGGCGTCGGTCTGCAGACCACCTTCGAGAAGCGCGTCGCTGTCCGTGCCGAAGTCGCCTACCGCGCTGATTTCGACGACCAGAGCGTGAACCCGAAGCGTGCCGGCAACGATGAAAGCTGGTTCGGCGACGTGCTGGCTTCGGTCGGCGTCGTGATCCCGCTGGGCCCGGCTCCGGTCGCGGCTGCTCCGGCTCCGGCTCCGGTTGCCCCGAGCTGCGCCGACCTGGATGACGACGGTGACGGCGTCAACAACTGCGACGACAAGTGCCCGAACTCGCAGCCGGGTCAGACCATCGGTCCGGACGGTTGCCCGGTGCCGGTCTCCATCGACCTGAAGGGCGTCAACTTCGACTTCGACAAGTCGAACCTGCGTCCGGACGCCGTGGCGATCCTGAGCGAAGCCACCGAGATCCTGAAGCGTTACCCGGATCTGCGCGTTGAAGTCGCCGGTCACACCGACTCGAAGGGTACCGACGCTTACAACCAGAAGCTGTCGGAGCGTCGTGCTACCGCCGTGTACAACTACCTGACCAAGAACGGCGTTGACGCCGGTCGCCTGGTCGGCCCGATCGGCTACGGCGAGAGCCGTCCGATTGCTCCGAACACCAACCCGGATGGTTCGGACAACCCGGAAGGTCGCGCCAAGAACCGTCGTACCGAGCTGAACGTCCAGAACTAA |
| outer membrane Omp family protein | Smlt4123 | WP_005411140.1 | ATGCGCTCCATCCGTATCCTGAGTCTCGCCCTGCTGACCTCCGCCGCCTTCGCGCCGGCCGCTTTCGCCCAGGACAGCAGCTCCACCGATACCGCTTCGGGCAAGCATTTTGCCGTGGTTGGCGGCGTCGCGCTGCTGCAGCCGAAGAATGATCCGATCGACGGCATCAAGAAGGTCGATGGTGGCCCGGCGCCGACCGTCAGCTTCAGCTACTACATCAACGACAACTGGGCCGTTGAACTGTGGGGCGCCGCCGACAAGTTCGACCACAAGGTGAAGGGCCCGAACAATGCCCGCCTGGGCAACGTCGAGCAGCAGCCGGTCGCGCTGAGCGGCCAGTACCACTTCGGCCAGGCTGACAACGTGTTCCGTCCGTTCGTGGGCGTGGGCTACTACCAGTCCAGCTTCAGCAATGAAACGCTGGCCGACGGCAGCAGCTCCGACATCCGCCTCAAGGACGCCAAGGGCGTGATCGGCACCGTCGGCGTGGACATGAACATCAACTCCACCTGGTTCGCCCGTGCCGATGCCCGCTACATGCGTTCGCGTCCGGACGTGAAGGTCGGCGGCGAGAAGATCGGCGAAGCCAAGATGGATCCGTGGACCGTCGGCTTCGGCATCGGTGCCCGCTTCTGA |

**Table S4 Determination of LD50**

| group | | dose (cfu per mouse) | mouse amount | death amount | LD50(cfu) |
| --- | --- | --- | --- | --- | --- |
| 1 | 1.2×10^9^ | | 8 | 8 | 5.8×10^8^ |
| 2 | 1.0×10^9^ | | 8 | 7 |  |
| 3 | 8.0×10^8^ | | 8 | 5 |  |
| 4 | 4.0×10^8^ | | 8 | 3 |  |
| 5 | 2.0×10^8^ | | 8 | 0 |  |



**Supplementary Figure 1**

**Figure S1. IgG1 and IgG2a levels in the pre-immune and post-immune mice injected with recombinant OmpA.**


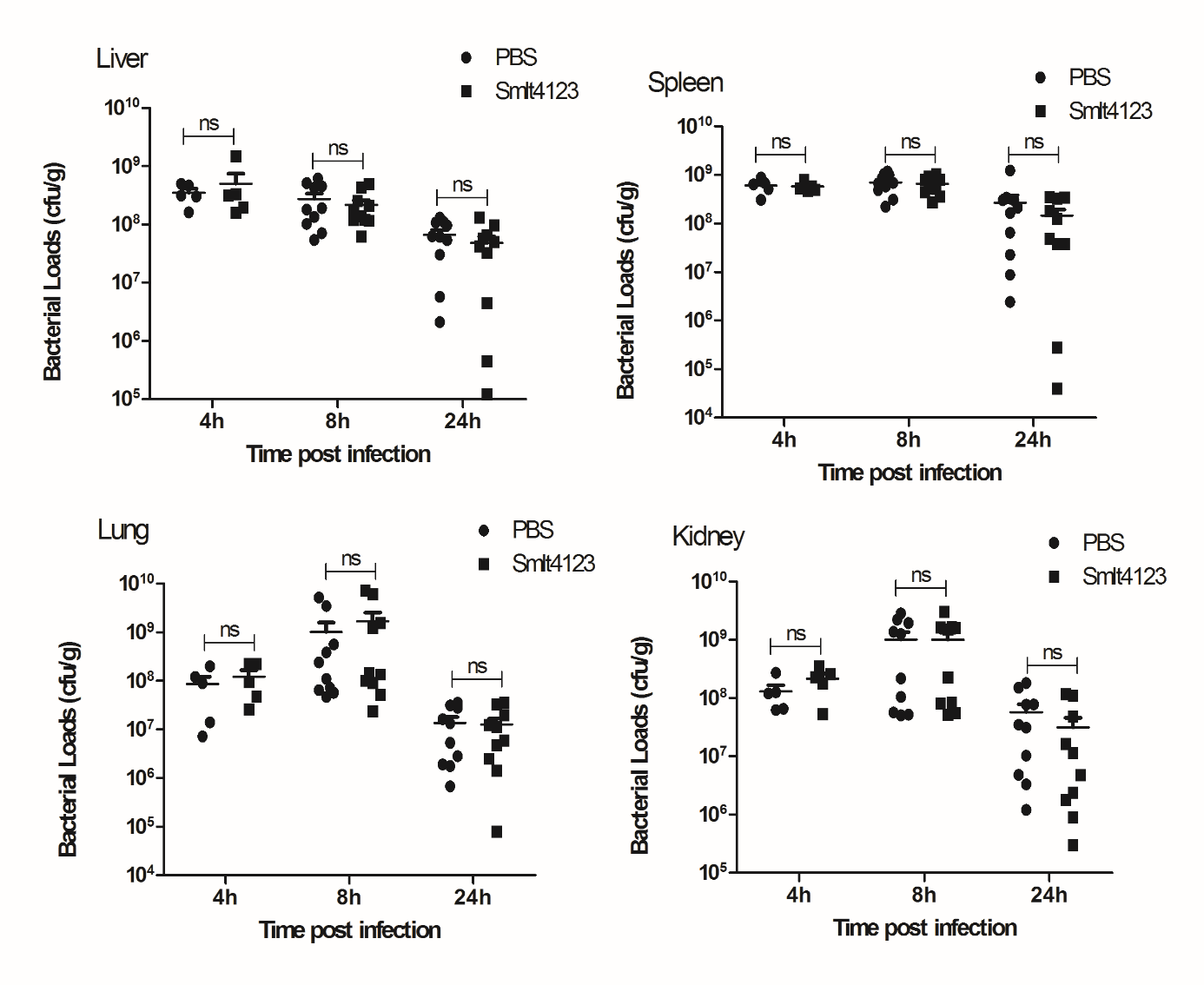
**Supplementary Figure 2**

**Figure S2.** **Bacterial loads in liver, spleen, lung, kidney of mice at different time points.**
